# Comparison of three rapamycin dosing schedules in A/J *Tsc2*^+/- ^mice and improved survival with angiogenesis inhibitor or asparaginase treatment in mice with subcutaneous tuberous sclerosis related tumors

**DOI:** 10.1186/1479-5876-8-14

**Published:** 2010-02-10

**Authors:** Chelsey Woodrum, Alison Nobil, Sandra L Dabora

**Affiliations:** 1 Translational Medicine Division, Department of Medicine, Brigham & Women's Hospital, Karp Building, Boston, MA, USA

## Abstract

**Background:**

Tuberous Sclerosis Complex (TSC) is an autosomal dominant tumor disorder characterized by the growth of hamartomas in various organs including the kidney, brain, skin, lungs, and heart. Rapamycin has been shown to reduce the size of kidney angiomyolipomas associated with TSC; however, tumor regression is incomplete and kidney angiomyolipomas regrow after cessation of treatment. Mouse models of *TSC2 *related tumors are useful for evaluating new approaches to drug therapy for TSC.

**Methods:**

In cohorts of *Tsc2*^+/- ^mice, we compared kidney cystadenoma severity in A/J and C57BL/6 mouse strains at both 9 and 12 months of age. We also investigated age related kidney tumor progression and compared three different rapamycin treatment schedules in cohorts of A/J *Tsc2*^+/- ^mice. In addition, we used nude mice bearing *Tsc2*^-/- ^subcutaneous tumors to evaluate the therapeutic utility of sunitinib, bevacizumab, vincristine, and asparaginase.

**Results:**

TSC related kidney disease severity is 5-10 fold higher in A/J *Tsc2*^+/- ^mice compared with C57BL/6 *Tsc2*^+/- ^mice. Similar to kidney angiomyolipomas associated with TSC, the severity of kidney cystadenomas increases with age in A/J *Tsc2*^+/- ^mice. When rapamycin dosing schedules were compared in A/J *Tsc2*^+/- ^cohorts, we observed a 66% reduction in kidney tumor burden in mice treated daily for 4 weeks, an 82% reduction in mice treated daily for 4 weeks followed by weekly for 8 weeks, and an 81% reduction in mice treated weekly for 12 weeks. In the *Tsc2*^-/- ^subcutaneous tumor mouse model, vincristine is not effective, but angiogenesis inhibitors (sunitinib and bevacizumab) and asparaginase are effective as single agents. However, these drugs are not as effective as rapamycin in that they increased median survival only by 24-27%, while rapamycin increased median survival by 173%.

**Conclusions:**

Our results indicate that the A/J *Tsc2*^+/- ^mouse model is an improved, higher through-put mouse model for future TSC preclinical studies. The rapamycin dosing comparison study indicates that the duration of rapamycin treatment is more important than dose intensity. We also found that angiogenesis inhibitors and asparaginase reduce tumor growth in a TSC2 tumor mouse model and although these drugs are not as effective as rapamycin, these drug classes may have some therapeutic potential in the treatment of TSC related tumors.

## Background

Tuberous Sclerosis Complex (TSC) is an autosomal dominant tumor disorder characterized by the manifestation of hamartomas in various organs including the kidney, brain, skin, lungs, and heart [1-3]. This multi-system disorder is fairly common, occurring at a frequency of 1:6000. The morbidity associated with TSC includes cognitive impairment, seizures, epilepsy, cortical tubers, cardiac rhabdomyomas, facial angiofibromas, and pulmonary lymphangioleiomyomatosis (LAM). Additionally, a majority of TSC patients experience renal manifestations such as kidney angiomyolipomas and/or kidney cysts. Kidney angiomyolipomas are age related tumors that occur in 60-80% of older children and adults with TSC [4,5] and approximately 50% of women with sporadic LAM [6]. Sporadic LAM is a progressive pulmonary disorder that is genetically related to TSC in that somatic mutations in the *TSC1 *or *TSC2 *genes have been identified in abnormal lung tissues from LAM patients [7].

TSC results from the loss of function of one of two genes, *TSC1 *or *TSC2*, whose gene products are hamartin and tuberin, respectively [8,9]. These two gene products form a tumor suppressor complex that functions to inhibit mTOR activity in a conserved cellular signaling pathway which is responsible for cell proliferation, protein synthesis, and nutrient uptake [10,11]. The key proteins in this pathway include PI3K, Akt, TSC1/TSC2, Rheb, and mTOR. The multiple roles of this important regulatory pathway have been described in recent reviews [12-16]. The inhibitory function of the tuberin-hamartin complex results from tuberin's GTP-ase activity on Rheb, which directly regulates mTOR kinase activity [17]. When conditions are unfavorable for cell growth and the TSC1/TSC2 complex is functioning properly, Rheb-GTP is converted to the GDP form and mTOR kinase activity is decreased. When mutations occur in *TSC1 *or *TSC2*, the hamartin-tuberin complex is nonfunctional, Rheb-GTP is favored, and mTOR kinase is constitutively activated causing hyperphosphorylation of the downstream effectors (p70 S6 kinase and 4E-binding protein1) resulting in increased protein translation, cell growth, proliferation, and survival.

Several TSC genotype-phenotype studies show that *TSC2 *disease is both more common and more severe than *TSC1 *disease [3,17-19]. The *Tsc2*^+/- ^mouse is a good model for TSC related kidney disease because it is genetically similar to the majority of those with TSC, it develops age related kidney tumors (cystadenomas), and the mTOR pathway defect that occurs in the kidney tumors of *Tsc2*^+/- ^mice is similar to that observed in human TSC related tumors [20-23]. Nude mice bearing subcutaneous *Tsc2*^-/- ^tumors derived from mouse embryo fibroblasts are another useful animal model for TSC related tumors. The *Tsc2*^-/- ^subcutaneous tumor model is a good generic model for TSC-related tumors because loss of heterozygosity (LOH) has been found in many TSC-related kidney and brain tumors [21,24,25].

Rapamycin (Rapamune™ or sirolimus, Wyeth, Madison, NJ) is a macrolide antibiotic that acts to inhibit the mTOR pathway and is FDA approved for use as an immunosuppressant following organ transplantation [26]. More recently, two rapamycin analogs (temsirolimus and everolimus) have been approved for the treatment of renal cell carcinoma [27,28]. Rapamycin (and analogs) have been shown to restore disregulated mTOR signaling in cells with abnormal TSC1 and/or TSC2 and to successfully treat kidney lesions in the *Tsc2*^+/- ^mouse model along with other rodent models [20,21,29-31]. Furthermore, in early clinical trials evaluating the utility of rapamycin for the treatment of kidney angiomyolipomas associated with TSC and/or LAM, partial tumor regression has been observed in the majority of cases. Because responses are incomplete, not all tumors respond to drug therapy, and patients experience kidney angiomyolipoma regrowth after cessation of treatment [32-34], further studies are needed to evaluate longer duration mTOR inhibitor treatment and also to identify other active drugs.

There is evidence that other drug classes, such as those that alter amino acid metabolism, inhibitors of VEGF signaling, and microtubule inhibitors may be useful in treating TSC. The presence or absence of amino acids is an important regulator of mTOR signaling [35]. L-Asparaginase is an enzyme that catalyzes the hydrolysis of L-asparagine to L-aspartic acid and is used as part of the curative combination chemotherapy regimen for the treatment of acute lymphoblastic leukemia (ALL) [36]. The anti-tumor effect of L-asparaginase is attributed to the depletion of the L-asparagine, but since some preparations have glutaminase activity, glutamine may also be depleted depending on the source of L-asparaginase. It has been shown that human leukemic cells treated with L-asparaginase have reduced levels of the mTOR pathway's targets p70 S6 kinase (p70^s6k^) and 4E-binding protein 1 (4E-BP1) [37]. Furthermore, there are tissue specific changes in mTOR pathway inhibition and cellular stress response signals in mice treated with L-asparaginase [38]. Due to its inhibitory effects on growth of malignant cells and mTOR pathway activity in some tissues, L-asparaginase may be useful in treating TSC related tumors.

Vascular endothelial growth factor (VEGF) signaling is thought to play an important role in the pathogenesis of TSC and LAM. Since the brain, skin, and kidney tumors associated with TSC are vascular [39] and TSC2 loss is associated with increased levels of HIF and VEGF in cultured cells [40], VEGF is a potential target for TSC treatment. Furthermore, recent studies have shown that serum VEGF-D levels are elevated in patients with sporadic or TSC-associated LAM compared with healthy controls and patients with other pulmonary ailments [41-43]. The importance of VEGF signaling in the pathogenesis of TSC suggests that VEGF inhibitors as single agents or in combination with mTOR inhibitors may provide a promising treatment. Sorafenib (also known as BAY 43-9006 and Nexavar) is an oral multi-targeted kinase inhibitor that blocks vascular endothelial growth factor receptor (VEGFR)-1, VEGFR-2, VEGFR-3, the RAF/Mek/Erk pathway, PDGFR, FLT-3, and C-KIT [44,45]. It is FDA approved for the treatment of advanced renal cell and hepatocellular carcinoma [46,47]. We have previously shown that the combination of sorafenib plus rapamycin is more effective than single agents in TSC tumor preclinical studies (Lee et al., 2009), but have not tested other VEGF signaling pathway inhibitors. Sunitinib (also known as SU11248 and Sutent) is a receptor tyrosine kinase inhibitor that targets both VEGF-R and platelet derived growth factor receptor (PDGF-R). Sunitinib has been shown to increase response and survival in patients with metastatic renal cell carcinoma (RCC) [48] and is also approved for the treatment of gastrointestinal stromal tumors [49]. Bevacizumab (also known as rhMAb-VEGF and Avastin) is a recombinant humanized monoclonal antibody that binds all human VEGF isoforms and is approved for the treatment of colon, breast, non-small cell lung cancer, and glioblastoma [50-54] and also prolongs the time to progression of disease in metastatic RCC [55,56]. The inhibitory effects of sunitinib and bevacizumab on VEGF signaling suggest that they may be useful in the treatment of TSC-related tumors.

Recent studies have shown that the TSC1/TSC2 complex may be important for microtubule-dependent protein transport because microtubule distribution and protein transport are disrupted in cells lacking *Tsc1 *or *Tsc2*. [57]. This raises the possibility that microtubule inhibitors may have useful anti-tumor activity for TSC related tumors. Vincristine is an anti-neoplastic microtubule inhibitor that binds tubulin dimers to arrest rapidly dividing cells in metaphase [58,59]. It is used in combination with other drugs in the treatment of lymphoma and leukemia. The defects in microtubule organization and function observed in *Tsc1 *and *Tsc2 *null cells suggests they may be sensitive to vincristine or other microtubule inhibitors.

In order to identify novel approaches for the treatment of tumors associated with TSC, we used two models of TSC related tumors in a series of preclinical studies. *Tsc2*^+/- ^mice were used to compare disease severity of kidney disease in two different mouse strains (C57BL/6 and A/J), evaluate the age related progression of kidney disease (in A/J mice), and compare three different dosing schedules of rapamycin (daily, daily plus weekly, and weekly). We used a subcutaneous *Tsc2*^-/- ^tumor model to evaluate the efficacy of two VEGF inhibitors (sunitinib and bevacizumab), asparaginase, and a microtubule inhibitor (vincristine).

## Methods

### Baseline tumor burden for untreated A/J versus C57BL/6 *Tsc2*^+/- ^mice and age related kidney disease in A/J *Tsc2*^+/- ^mice

The *Tsc2*^+/- ^mouse is heterozygous for a deletion of exons 1-2 as previously described [60]. In order to determine the baseline tumor burden for untreated *Tsc2*^+/- ^in the A/J and C57BL/6 backgrounds, strain specific colonies of each background were created. Strain specific colonies were created for both the A/J and C57BL/6 background by backcrossing female *Tsc2 *heterozygous offspring with their pure strain *Tsc2 *wildtype fathers until the N5 generation was reached. Mice from the N5 generations were assigned to cohorts based on age, gender, and genotype. The cohorts were: *Tsc2*^+/- ^9 months consisting of 8 males and 8 females, *Tsc2*^+/+ ^9 months consisting of 2 males and 2 females, *Tsc2*^+/- ^12 months consisting of 4 males and 4 females, and *Tsc2*^+/+ ^12 months consisting of 2 males and 2 females. To determine the age related kidney disease in the A/J background, A/J *Tsc2*^+/- ^mice were assigned to three additional cohorts. The cohorts were: A/J *Tsc2*^+/- ^3 months, A/J *Tsc2*^+/- ^5 months, and A/J *Tsc2*^+/- ^7 months. Each cohort contained 4 mice.

Mice were sacrificed according to age and cohort assignment. Upon sacrifice, kidneys, livers, and lungs were examined. All animals in *Tsc2*^+/- ^cohorts had gross kidney lesions. There were no obvious liver tumors. Three A/J *Tsc2*^+/- ^animals had gross lung abnormalities (1 in the untreated 3 month cohort, and 2 in the cohort treated with weekly rapamycin × 12 weeks) and one mouse, from the cohort treated with weekly rapamycin × 12 weeks, had a superficial tail tumor. Since non-kidney tumors were rare events, these were not studied further. We also looked at *Tsc2*^+/+ ^cohorts at nine and twelve months of age and observed no gross or microscopic kidney lesions.

### Quantification of kidney cystadenomas in *Tsc2*^+/- ^mice

For histological quantification of kidney cystadenomas, each kidney was prepared as previously described [61]. All cystadenomas were counted, measured, and scored according to the scale shown in Additional File 1 by a blinded researcher (CW or AN). Since the kidney cystadenomas of these *Tsc2*^+/- ^mice can be divided into the subgroups cystic, pre-papillary, papillary and solid lesions, we use "kidney cystadenomas" to refer to the entire spectrum of kidney lesions observed. In addition to analyzing data according to all cystadenomas, a subgroup analysis was also done by coding cystic, pre-papillary, papillary, and solid kidney lesions separately. The scale used to define cystadenoma subtypes is shown in Additional File 2.

### Rapamycin dosing schedules in A/J *Tsc2*^+/- ^mice

A/J *Tsc2*^+/- ^mice were assigned to one of three different rapamycin treatment cohorts (Groups 1-3) or an untreated control group (Group 4). The rapamycin cohorts included the following schedules: daily × 4 weeks plus weekly × 8 weeks (Group 1), daily × 4 weeks (Group 2), weekly × 12 weeks (Group 3). All animals started treatment at nine months of age and were euthanized twelve weeks later. Mice in Group 1 were treated with 8 mg/kg rapamycin administered by intraperitoneal injection (IP) Monday through Friday for four weeks followed by weekly doses of 8 mg/kg rapamycin IP for eight weeks. Mice in Group 2 were treated with 8 mg/kg rapamycin IP Monday through Friday for four weeks and received no drug treatment for the next 8 weeks. Mice in Group 3 were treated with weekly 8 mg/kg rapamycin IP for twelve weeks. Rapamycin powder was obtained from LC Laboratories (Woburn, MA) and a 20 mg/ml stock of rapamycin was made in ethanol (stored at -20°C for up to one week). The stock solution was diluted to 1.2 mg/ml in vehicle (0.25% PEG, 0.25% Tween-80) for the 8 mg/kg dose. Rapamycin treatments were administered within two hours of their preparation. All animals were checked daily (5 days per week), and general health and behavior were noted. All rapamycin treated animals were weighed at 9 months (at the start of rapamycin treatment), and again at the time of euthanasia at ~12 months (see Additional File 3). All mice were euthanized at approximately twelve months of age according to institutional animal care guidelines. The severity of kidney disease was calculated using quantitative histopathology as described previously. Untreated A/J *Tsc2*^+/- ^mice from the 9 month and 12 month cohorts were weighed at the time of necropsy for comparison. All experiments were done according to animal protocols approved by our institutional animal protocol review committee (Children's Hospital Boston, Boston, MA) and were compliant with federal, local, and institutional guidelines on the care of experimental animals.

### Treatment of subcutaneous tumors with asparaginase, vincristine, sunitinib, bevacizumab, and rapamycin

Nude mice (strain CD-1nuBR, up to 6-8 weeks old) were obtained from Charles River Laboratories, Inc. (Wilmington, Massachusetts) and injected subcutaneously on the dorsal flank with 2.5 million NTC/T2null (*Tsc2*^-/-^*, Trp53*^-/-^) cells. NTC/T2null cells are mouse embryonic fibroblasts that have been described previously [21]. A total of 80 CD-1 nude mice were divided into 10 randomly assigned groups: untreated control group, single agent rapamycin, single agent asparaginase, combination asparaginase plus rapamycin, single agent vincristine, combination vincristine plus rapamycin, single agent sunitinib, combination sunitinib plus rapamycin, single agent bevacizumab, and combination bevacizumab plus rapamycin. As soon as tumors became visible, they were measured Monday through Friday using calipers. Tumor volumes were calculated using the formula: length × width × width × 0.5. All mice began treatment when tumors reached a volume of ~100 mm^3^. All mice were euthanized once tumors reached ~3000 mm^3 ^in accordance with institutional animal care guidelines.

Untreated mice did not receive any treatment even after tumors reached a volume ≥ 100 mm^3^. Rapamycin treated groups received 200 μl of a 1.2 mg/ml solution of rapamycin (8 mg/kg) three times per week (on Mondays, Wednesdays, and Fridays) by IP injection. Doses of asparaginase, vincristine, sunitinib, and bevacizumab were selected based on anti-tumor activity in published preclinical studies [38,62-64]. Asparaginase treated groups received 200 μl of a 300 IU/mL solution of asparaginase on Mondays and Thursdays for 4 weeks by IP injection. Vincristine treated groups received 200 μl of a 0.075 mg/mL solution of vincristine once per week for four weeks by IP injection. Sunitinib treated groups received 200 μl of a 12 mg/mL solution of sunitinib daily (Monday-Friday) by gavage. Bevacizumab treated groups received 200 μl of 0.75 mg/mL solution of bevacizumab once every two weeks by IP injection. All drug doses were calculated assuming a weight of 30 g per mouse. Asparaginase powder was obtained from the Brigham and Women's Hospital Research Pharmacy (Boston, MA) and diluted in sterile PBS. Vincristine was obtained in a 1 mg/mL solution from the Brigham and Women's Hospital Research Pharmacy (Boston, MA) and diluted in sterile PBS. Bevacizumab was obtained in a 25 mg/mL solution from the Brigham and Women's Hospital Research Pharmacy (Boston, MA) and diluted in sterile phosphate buffered saline (PBS). Sunitinib powder was obtained from LC Laboratories (Woburn, MA) and diluted in a sterile 5% glucose solution. Rapamycin powder was obtained from LC Laboratories (Woburn, MA) and a 20 mg/mL stock of rapamycin was made in ethanol (stored at -20°C for up to one week). The stock solution was diluted to 1.2 mg/mL in vehicle (0.25% PEG-400, 0.25% Tween-80).

Animal behavior and health were monitored daily, and animals were weighed at the start of the study and at the time of necropsy. Six animals had to be euthanized early due to dehydration and weight loss (Additional File 4). The survival and tumor growth data for these animals were included in all analyses. All mice from rapamycin treated cohorts were euthanized 24 hours after the last rapamycin treatment upon reaching the endpoint tumor volume. Upon sacrifice, whole blood was obtained for drug level testing.

### Whole blood rapamycin levels

Whole blood rapamycin levels were measured from a subset of animals treated with rapamycin in the nude mouse treatment studies described above. Blood was removed at necropsy 24 hours after the final treatment of rapamycin. Whole blood was obtained through cardiac puncture, dispensed into an EDTA-containing blood collection tube, and diluted with an equal volume of sterile PBS to ensure sufficient volume for rapamycin level analysis. All measured rapamycin levels were corrected according to sample dilution at time of analysis. Only bevacizumab plus rapamycin, sunitinib plus rapamycin and single agent rapamycin cohorts could be analyzed for rapamycin levels due to treatment schedules. Whole blood samples were tested for rapamycin levels at the Clinical Laboratory at Children's Hospital Boston (Boston, Massachusetts). The range of detection is 0.5 to 100 ng/ml of rapamycin.

### Statistical analyses

GraphPad Prism software (version 4.01) was used for all data analysis, with a p-value ≤ 0.05 indicating statistical significance. All calculations were completed from raw data by two researchers (AN and CW). A standard unpaired *t *test was used to test all quantitative data, and the Mantel-Cox logrank analysis was used for survival data.

## Results

### Kidney tumor severity is age related and increased in A/J *Tsc2*^+/- ^mice compared with C57BL/6 *Tsc2*^+/- ^mice

**Figure 1:**
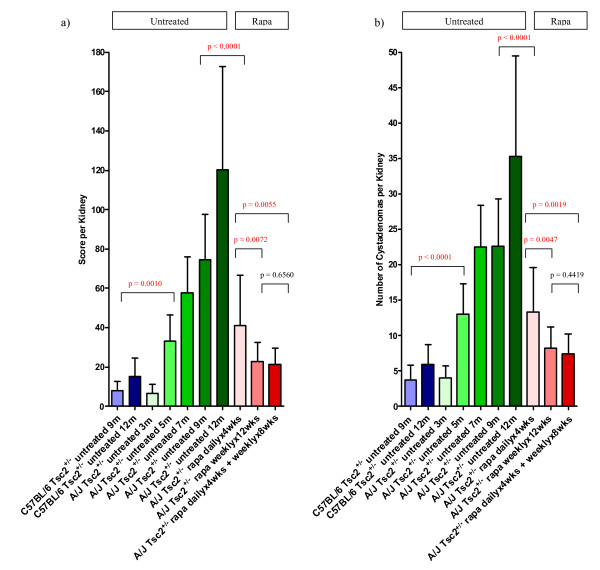
**A/J strain *Tsc2*^+/- ^mice show an increased severity of kidney disease with age, a greater kidney tumor burden than C57BL/6 *Tsc2*^+/- ^mice, and best response to longer duration rapamycin treatment**. The average score per kidney for each cohort is shown in 1a. The average number of cystadenomas per kidney for each cohort is shown in 1b. The red p-values indicate a statistically significant difference (p < 0.05) between the two cohorts being compared. These data show a significant increase in both the score per kidney and the number of cystadenomas per kidney in the A/J strain as compared to the C57BL/6 strain for both 9 months of age and 12 months of age. Additionally, these data show a significant increase with age in both the score per kidney and the number of cystadenomas per kidney for the A/J *Tsc2*^+/- ^strain. Furthermore, the tumor burden is reduced with rapamycin therapy with the weekly × 12 weeks cohort and the daily × 4 weeks plus weekly × 8 weeks cohort showing the most reduction. This data is summarized in Table 1.

In order to compare kidney disease severity in different *Tsc2*^+/- ^mouse strains, we evaluated kidney cystadenomas in cohorts of A/J and C57BL/6 *Tsc2*^+/- ^mice at nine and twelve months of age. Kidney disease severity for all cohorts is shown in Figure 1 and Table 1. Untreated A/J cohorts are shown in green, and untreated C57BL/6 cohorts are shown in blue. Although data are shown as both average cystadenoma score per kidney (Figure 1a) and average number of cystadenomas per kidney (Figure 1b), these have a similar trend. The average score per kidney for the A/J *Tsc2*^+/- ^untreated 12 m cohort (120.20 ± 52.53) is significantly greater (p < 0.0001) than that of the C57BL/6 *Tsc2*^+/- ^untreated 12 m cohort (15.19 ± 9.39). Similarly, the average score per kidney for the A/J *Tsc2*^+/- ^untreated 9 m cohort (74.47 ± 23.07) is significantly greater (p < 0.0001) than that of the C57BL/6 *Tsc2*^+/- ^untreated 9 m cohort (7.97 ± 4.76). Interestingly, the average score per kidney for the A/J *Tsc2*^+/- ^untreated 9 m cohort is significantly greater (p < 0.0001) than that of the C57BL/6 *Tsc2*^+/- ^untreated 12 m cohort. Since A/J *Tsc2*^+/- ^mice have a higher average score per kidney at nine months of age than C57BL/6 *Tsc2*^+/- ^mice at 12 months of age, these data show that the A/J *Tsc2*^+/- ^strain has a significantly higher tumor burden than the C57BL/6 *Tsc2*^+/- ^strain. There is no significant difference in severity of kidney disease between males and females within the same strain (see Additional File 5). This is true for both A/J *Tsc2*^+/- ^mice and C57BL/6 *Tsc2*^+/- ^mice at 9 months of age and 12 months of age.

**Table 1:** Average Score and Number of Cystadenomas per Kidney for A/J and C57BL/6 *Tsc2*^+/- ^Cohorts

*Tsc2*^*+/- *^Cohort (strain, treatment, age)	Score per Kidney (ave ± std dev)	Number per Kidney (ave ± std dev)	% Reduction in Score per Kidney vs. Group 4	n	Group Number	Number of Rapa Doses	Duration of Treatment	Total Dose per Mouse (mg)
C57BL/6, untreated, 12 months	15.19 ± 9.39	5.94 ± 2.79		8				

A/J, untreated, 3 months	6.50 ± 4.60	4.00 ± 1.69		4				

A/J, untreated, 5 months	33.00 ± 13.53	13.00 ± 4.28		4				

A/J, untreated, 7 months	57.75 ± 18.24	22.50 ± 5.88		4				

A/J, untreated, 9 months	74.47 ± 23.07	22.63 ± 6.66		16				

**A/J, untreated, 12 months	120.20 ± 52.53	35.25 ± 14.22		8	4			

Group 1 *A/J rapa daily × 4 weeks then weekly × 8 weeks	21.50 ± 8.38	7.38 ± 2.83	82%	8	1	28	12 weeks	6.72

Group 2 *A/J rapa daily × 4 weeks	41.13 ± 25.33	13.25 ± 6.32	66%	8	2	20	4 weeks	4.8

Group 3 *A/J rapa weekly × 12 weeks	22.61 ± 9.89	8.17 ± 3.07	81%	9	3	12	12 weeks	2.88

* All treatments started at 9 months of age, and mice were euthanized 12 weeks later (at ~12 months of age)

** Untreated controls were euthanized at 12 months of age

From previous studies, we have shown that the severity of kidney disease increases with age in C57BL/6 *Tsc2*^+/- ^mice [20]. In order to understand the progression of kidney tumor growth in A/J *Tsc2*^+/- ^mice, data was collected at different time points. The average score per kidney for the A/J *Tsc2*^+/- ^mice at 3 months, 5 months, and 7 months of age was 6.5, 33.0, and 57.7, respectively. It is important to note that the score per kidney for the A/J *Tsc2*^+/- ^untreated 5 m cohort (33.00 ± 13.53) is significantly greater (p = 0.0010) than that of the C57BL/6 *Tsc2*^+/- ^untreated 12 m cohort (15.19 ± 9.39). These data further confirm that the A/J *Tsc2*^+/- ^strain develops more severe kidney disease than the C57BL/6 *Tsc2*^+/- ^strain and will allow for higher through-put *Tsc2*^+/- ^preclinical studies.

### Comparison of three rapamycin dosing schedules in *Tsc2*^+/- ^mice

In a prior preclinical study, we determined that daily rapamycin treatment for two months combined with a rapamycin maintenance dose once a week for five months dramatically reduced tumor burden by 94.5% as compared to the untreated control [61]. However, because that study included only one single agent rapamycin treatment group in which animals were treated daily × 1 month, then weekly × 4 months, then daily × 1 month, we do not clearly understand the impact of weekly rapamycin treatment. In order to further evaluate the efficacy of rapamycin weekly maintenance dosing, here we compared three rapamycin dosing schedules in A/J *Tsc2*^+/- ^mice (weekly, daily, daily plus weekly). All animals started treatment at 9 months of age and were euthanized 12 weeks after treatment started. As shown in Table 1 and Figure 1, all three treatment cohorts showed a significant decrease in the average cystadenoma score per kidney as compared to both the 9 month and 12 month A/J *Tsc2*^+/- ^untreated control groups (number of cystadenomas gave similar trends). Additionally, rapamycin dosed daily × 4 weeks followed by weekly × 8 weeks (Group 1, score per kidney 21.5) was more effective than rapamycin dosed daily × 4 weeks with no weekly maintenance dosing (Group 2, score per kidney 41.1, p = 0.007).

This data indicates that there was some tumor regrowth during the 8 weeks off of treatment in Group 2. Interestingly, dosing rapamycin weekly × 12 weeks (Group 3, score per kidney 22.6) was equally effective compared with dosing rapamycin daily × 4 weeks plus weekly × 8 weeks (Group 1). This suggests that the duration of rapamycin exposure is the critical factor and dose intensity is less important as there was no benefit to giving the higher doses for the first 4 weeks in Group 1. According to drug level testing in whole blood for this and prior preclinical studies [20,65], average rapamycin levels in whole blood are ~12-40 ng/ml from 24 hours to 6 days, and ~6 ng/ml on days 7-8 after a single 8 mg/kg dose. This indicates that weekly rapamycin dosing in mice correlates well with clinical dosing in humans for which the typical range for target trough (24 hour) levels is 3-20 ng/ml.

### Kidney cystadenoma subtypes are similar in A/J and C57BL/6 cohorts and shift to more pre-papillary and cystic lesions with rapamycin treatment

**Figure 2:**
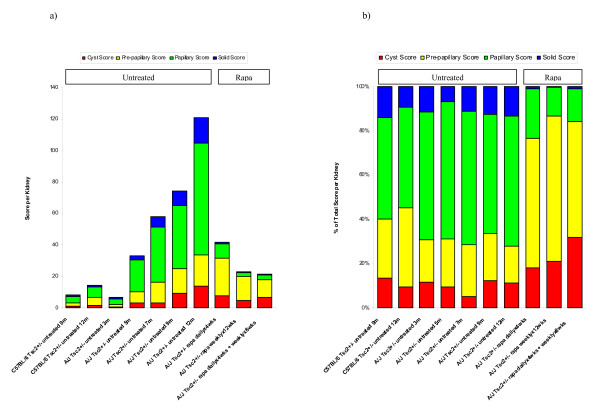
**Rapamycin treated *Tsc2*^+/- ^mice show a higher percentage of cystic and pre-papillary cystadenomas and a smaller percentage of papillary and solid cystadenomas**. The absolute score per kidney for each cystadenoma subtype is shown in Figure 2a, and the percent of total score per kidney for each cystadenoma subtype is shown in Figure 2b. For a description of each subtype, see Additional File 2. Papillary cystadenomas contribute the largest percentage to total score per kidney in untreated A/J and C57BL/6 cohorts at all time points. Pre-papillary cystadenomas contribute the largest percentage to total score per kidney in A/J cohorts treated with rapamycin. Treatment with rapamycin results in a decrease in the percentages of papillary and solid cystadenomas and an increase in the percentages of pre-papillary and cystic cystadenomas.

We determined kidney cystadenoma subtypes for all A/J and C57BL/6 cohorts. The total score per kidney categorized by each cystadenoma subtype is shown in Figure 2a, and the percent contribution to total score per kidney for each cystadenoma subtype is shown in Figure 2b and Table 2. For all of the A/J and C57BL/6 untreated cohorts, papillary lesions contributed the greatest percentage to total score per kidney while cystic and solid lesions account for the smallest percentage. Papillary lesions made up 53-62% of the total score per kidney for the A/J untreated cohorts and 43-46% for the C57BL/6 untreated cohorts. Cystic lesions made up 5-12% of the total score per kidney for the A/J untreated cohorts and 9-13% for the C57BL/6 untreated cohorts. Pre-papillary lesions contributed 17-24% to the total score per kidney for the A/J untreated cohorts and 26-34% for the C57BL/6 untreated cohorts. Solid lesions contributed 7-14% to the total score per kidney for the A/J untreated cohorts and 9-14% for the C57BL/6 untreated cohorts. Compared to the untreated control cohorts, all rapamycin treatment cohorts showed a lower percentage of papillary (13-23%) and solid (0-1%) lesions and a higher percentage of cystic (18-31%) and pre-papillary (51-66%) lesions. These data suggest that rapamycin treatment may cause a shift from solid and papillary cystadenomas to cystic and pre-papillary cystadenomas.

**Table 2:** Distribution of Kidney Lesion Subtype for A/J and C57BL/6 *Tsc2*^+/- ^Cohorts

	% of Total Score per Kidney			
	
*Tsc2*^**+/- **^Cohort (strain, treatment, age)	Cyst	Pre-papillary	Papillary	Solid
C57BL/6, untreated, 9 months	13.34	26.67	45.88	14.11

C57BL/6, untreated, 12 months	8.64	34.15	43.21	8.64

A/J, untreated, 3 months	11.54	19.23	57.69	11.54

A/J, untreated, 5 months	9.47	21.59	62.12	6.82

A/J, untreated, 7 months	4.98	23.6	60.17	11.26

A/J, untreated, 9 months	12.38	21.27	53.63	12.51

A/J, untreated, 12 months	11.18	16.75	59.07	13.52

Group 1 A/J rapa daily × 4 weeks then weekly × 8 weeks	31.4	51.44	14.83	0.87

Group 2 A/J rapa daily × 4 weeks	18.08	58.67	22.64	0.91

Group 3 A/J rapa weekly × 12 weeks	20.88	65.86	13.02	0.25

### Treatment of *Tsc2*^-/- ^subcutaneous tumors with angiogenesis inhibitors, asparaginase, and vincristine

**Figure 3:**
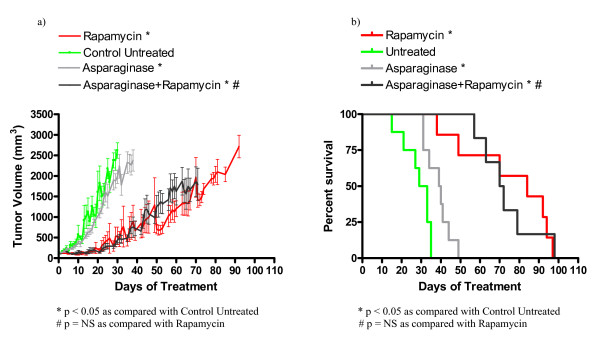
**Asparaginase treatment improved survival and decreased tumor growth in nude mice bearing *Tsc2*^-/- ^tumors**. (a) Average tumor volume over time for asparaginase and asparaginase plus rapamycin treated animals. (b) Survival curve for indicated treatment cohorts. Based on survival analysis and comparison of tumor volumes on day 30, asparaginase improves survival and decreases tumor growth compared to the untreated cohort. Asparaginase is not as effective as single agent rapamycin in improving survival or decreasing tumor growth. Based on analysis and comparisons of tumor volumes on day 65, asparaginase in combination with rapamycin provided no improvement over single agent rapamycin treatment.

**Figure 4:**
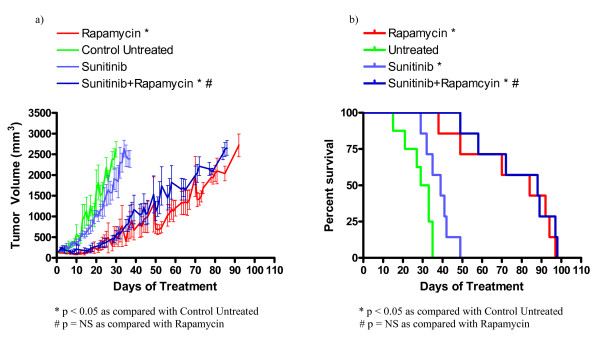
**Sunitinib treatment improved survival in nude mice bearing *Tsc2*^-/- ^tumors**. (a) Average tumor volume over time for sunitinib and sunitinib plus rapamycin treated animals. (b) Survival curve for indicated treatment cohorts. Based on survival analysis and comparison of tumor volumes on day 30, sunitinib improves survival but does not decrease tumor growth compared to the untreated cohort. Sunitinib is not as effective as single agent rapamycin in improving survival or decreasing tumor growth. Based on analysis and comparisons of tumor volumes on day 65, sunitinib in combination with rapamycin provided no improvement over single agent rapamycin treatment.

**Figure 5:**
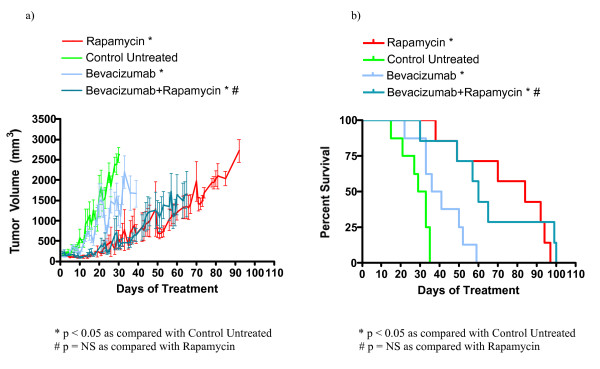
**Bevacizumab treatment improved survival and decreased tumor growth in nude mice bearing *Tsc2*^-/- ^tumors**. (a) Average tumor volume over time for bevacizumab and bevacizumab plus rapamycin treated animals. (b) Survival curve for indicated treatment cohorts. Based on survival analysis and comparison of tumor volumes on day 30, bevacizumab improves survival and decreases tumor growth compared to the untreated cohort. Bevacizumab is not as effective as single agent rapamycin in improving survival or decreasing tumor growth. Based on analysis and comparisons of tumor volumes on day 65, bevacizumab in combination with rapamycin provided no improvement over single agent rapamycin treatment.

**Figure 6:**
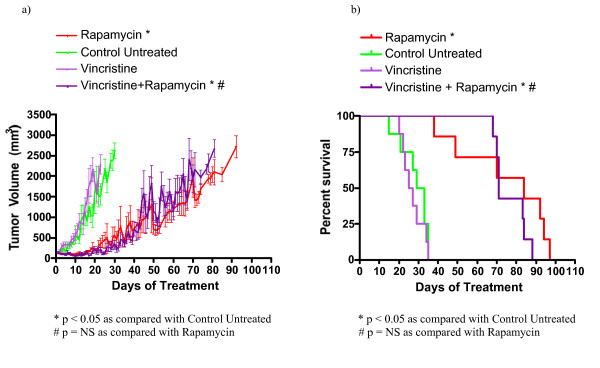
**Vincristine does not decrease tumor growth or increase survival in nude mice bearing *Tsc2*^-/- ^tumors**. (a) Average tumor growth over time for vincristine and vincristine plus rapamycin treated animals. (b) Survival curve for indicated cohorts. Based on survival analysis and comparison of tumor volumes on days 23 and 65, vincristine was not effective as a single agent or in combination with rapamycin.

In order to evaluate the utility of some novel drug classes for the treatment of TSC related tumors, we investigated the efficacy of asparaginase, sunitinib, bevacizumab, and vincristine in treating a relevant subcutaneous tumor model. We used nude mice bearing subcutaneous *Tsc2*^-/- ^tumors derived from NTC/T2 null cells in a preclinical study with the following cohorts: untreated, rapamycin treated, asparaginase treated, asparaginase plus rapamycin combination treated, vincristine treated, vincristine plus rapamycin combination treated, sunitinib treated, sunitinib plus rapamycin treated, bevacizumab treated, and bevacizumab plus rapamycin treated. Average tumor growth for each cohort is shown in Figures 3a, 4a, 5a, 6a, and Table 3. The data points represent days when at least four mice of the treatment group had tumors measured. Tumor volumes for single agents were compared to untreated controls on day 30 for all groups except vincristine because this was the last day with at least four data points for the untreated group; day 23 was used for vincristine (last day with at least four data points). Tumor volumes for combination treatments were compared to single agent rapamycin treatment on day 65 because this was the last day with at least four data points for all combination treatment groups. Survival curves for each cohort are shown in Figures 3b, 4b, 5b, and 6b. Survival curves were compared using the Mantel Cox logrank analysis.

**Table 3:** Summary of *Tsc2*-/- Subcutaneous Tumor Data (Vincristine, Asparaginase, Sunitinib, and Bevacizumab)

	Untreated	Rapamycin	Vincristine	Combination Vincristine plus Rapamycin	Asparaginase	Combination Asparaginase plus Rapamycin	Sunitinib	Combination Sunitinib plus Rapamycin	Bevacizumab	Combination Bevacizumab plus Rapamycin
Number of mice (n)	8	8	8	8	8	8	8	8	8	8

Median Survival (days)	31	84.5	26	77	39.5	71	39	80	38.5	60
P value (survival)	-	<0.0001*	NS*	NS^#^	0.0101*	NS^#^	0.0193*	NS^#^	0.0131*	NS^#^

Day 23, average tumor volume ± SEM (mm^3^)	1557 ± 260	352 ± 149	2289 ± 242	-	-	-	-	-	-	-
P Value (Day 23)	-	0.0016*	NS*	-	-	-	-	-	-	-

Day 30, average tumor volume ± SEM (mm^3^)	2618 ± 187	545 ± 212	-	330 ± 101	1978 ± 167	441 ± 97	1886 ± 287	545 ± 114	1233 ± 366	813 ± 449
P Value (Day 30)	-	0.0001*	-	-	0.0405*	-	NS*	-	0.0172*	-

Day 65, average tumor volume ± SEM (mm^3^)	-	1349 ± 302	-	2050 ± 384	-	1570 ± 378	-	1643 ± 246	-	1652 ± 557
P Value (Day 65)	-	-	-	NS^#^	-	NS^#^	-	NS^#^	-	NS^#^

Rapamycin (IP, 3 days per week)	-	8 mg/kg, 3 days per week	-	8 mg/kg, 3 days per week	-	8 mg/kg, 3 days per week	-	8 mg/kg, 3 days per week	-	8 mg/kg, 3 days per week

Vincristine (IP, weekly × 4 weeks)	-	-	0.5 mg/kg, weekly × 4 weeks	0.5 mg/kg, weekly × 4 weeks	-	-	-	-	-	-

Asparaginase (IP, Mon, Thurs × 4 weeks)	-	-	-	-	2 IU/g, Mon, Thurs × 4 weeks	2 IU/g, Mon, Thurs × 4 weeks	-	-	-	-

Sunitinib (Gavage, Mon-Fri)	-	-			-	-	80 mg/kg, Mon- Fri	80 mg/kg, Mon- Fri	-	-

Bevacizumab (IP, once/2 weeks)	-	-	-	-	-	-	-	-	5 mg/kg, once/2 weeks	5 mg/kg, once/2 weeks

* compared to untreated

# compared to rapamycin treated

NS, not significant

Single agent asparaginase improves survival and reduces *Tsc2*^-/- ^tumor growth. The day 30 average tumor volume for the asparaginase cohort (1978 ± 167 mm^3^) and the untreated cohort (2618 ± 187 mm^3^) are significantly different (p = 0.0405). The average tumor volumes at day 65 for the asparaginase plus rapamycin cohort (1570 ± 378 mm^3^) and the rapamycin cohort (1349 ± 302 mm^3^) are similar (Figure 3a, Table 3). The median survival of the single agent asparaginase cohort (39.5 days) and the median survival of the untreated cohort (31 days) are significantly different (p = 0.0101). However, the median survival of the asparaginase plus rapamycin treated cohort (71 days) is not significantly different than the median survival of the single agent rapamycin treated cohort (84.5 days, Figure 3b, Table 3). The slightly lower median survival in the asparaginase plus rapamycin combination group suggests that adding asparaginase to rapamycin may enhance tumor growth in some cases, although the mechanism is not known. In summary, asparaginase as a single agent is effective at reducing tumor growth and increasing survival when compared to the untreated cohort. Single agent asparaginase is not as effective as rapamycin at decreasing tumor volume or increasing survival. Furthermore, adding asparaginase to rapamycin did not reduce disease severity when compared to single agent rapamycin.

Single agent sunitinib improves survival in mice bearing *Tsc2*^-/- ^tumors. The day 30 average tumor volume for the sunitinib cohort (1886 ± 287 mm^3^) was smaller than that of the untreated cohort (2618 ± 187 mm^3^), but this difference was not statistically significant. The average tumor volumes at day 65 for the sunitinib plus rapamycin cohort (1643 ± 246 mm^3^) and the rapamycin cohort (1349 ± 302 mm^3^) are similar (Figure 4a, Table 3). The median survival of the single agent sunitinib cohort (39 days) and the median survival of the untreated cohort (31 days) are significantly different (0.0193). However, the median survival of the sunitinib plus rapamycin treated cohort (80 days) is not significantly different than the median survival of the single agent rapamycin treated cohort (84.5 days, Figure 4b, Table 3). In summary, sunitinib as a single agent is effective at increasing survival, but not at reducing tumor growth, when compared to the untreated cohort. Single agent sunitinib is not as effective as rapamycin at decreasing tumor volume or increasing survival. Furthermore, adding sunitinib to rapamycin did not reduce disease severity when compared to single agent rapamycin.

Single agent bevacizumab improves survival and reduces *Tsc2*^-/- ^tumor growth. The day 30 average tumor volume for the bevacizumab cohort (1233 ± 366 mm^3^) and the untreated cohort (2618 ± 187 mm^3^) are significantly different (p = 0.0172). The average tumor volumes at day 65 for the bevacizumab plus rapamycin cohort (1652 ± 557 mm^3^) and the rapamycin cohort (1349 ± 302 mm^3^) are similar (Figure 5a, Table 3). The median survival of the single agent bevacizumab cohort (38.5 days) and the median survival of the untreated cohort (31 days) are significantly different (p value = 0.0131). However, the median survival of the bevacizumab plus rapamycin treated cohort (60 days) is not significantly different than the median survival of the single agent rapamycin treated cohort (84.5 days, Figure 5b, Table 3). The slightly lower median survival in the bevacizumab plus rapamycin combination group suggests that adding bevacizumab to rapamycin may enhance tumor growth in some cases, although the mechanism is not known. In summary, bevacizumab as a single agent is effective at reducing tumor growth and increasing survival when compared to the untreated cohort. Single agent bevacizumab is not as effective as rapamycin at decreasing tumor volume or increasing survival. Furthermore, adding bevacizumab to rapamycin did not reduce disease severity when compared to single agent rapamycin.

Vincristine was not effective for the treatment of *Tsc2*^-/- ^tumors. The day 23 average tumor volume for the vincristine cohort (2289 ± 242 mm^3^) and the untreated cohort (1557 ± 260 mm^3^) are not significantly different. The average tumor volumes at day 65 for the vincristine plus rapamycin cohort (2050 ± 384 mm^3 ^and the rapamycin cohort (1349 ± 302 mm^3^) are similar. (Figure 6a, Table 3). Survival data shows that the median survival of the single agent vincristine cohort (26 days) does not differ significantly from the median survival of the untreated cohort (31 days). The median survival of the vincristine plus rapamycin treated cohort (77 days) is also not significantly different than the median survival of the single agent rapamycin treated cohort (84.5 days, Figure 6b, Table 3). In summary, vincristine as a single agent is not effective at reducing tumor growth and increasing survival when compared to the untreated cohort or the single agent rapamycin cohort. Furthermore, adding vincristine to rapamycin did not reduce disease severity when compared to single agent rapamycin.

### Rapamycin drug levels in combination treated animals

Rapamycin is metabolized by CYP3A4 therefore drug levels can vary when there is exposure to other drugs that either induce or inhibit CYP3A4. To be sure there were no significant drug interaction issues in our studies, rapamycin levels were measured in tumors or whole blood 24 hours after the last dose in a subset of animals from our studies (Additional File 6). Average blood rapamycin levels in the sunitinib plus rapamycin group (137.9 ± 29.23 ng/ml), bevacizumab plus rapamycin group (94 ± 34.4 ng/ml), and the single agent rapamycin group (86.4 ± 0.86 ng/ml) were not statistically different. Rapamycin levels for the asparaginase plus rapamycin and vincristine plus rapamycin cohorts are not reported due to the treatment schedules of asparaginase and vincristine. Asparaginase and vincristine treatments were given for only 4 weeks and so had not been administered to mice in these cohorts for several weeks prior to the last dose of rapamycin. Based on drug level testing, we conclude that sunitinib and bevacizumab did not significantly affect the metabolism of rapamycin in the preclinical studies reported here.

### Rapamycin treatment associated with lack of weight gain in nude mice bearing *Tsc2*^-/- ^tumors

Six rapamycin treated nude mice bearing *Tsc2*^-/- ^subcutaneous tumors required early euthanasia. The six mice presented with hunched posture, dehydration, and weight loss, and were euthanized per protocol standards. Each of the six mice belonged to different treatment cohorts; however, all of the mice received rapamycin treatment (Additional File 4). Because nude mice are immunodeficient and rapamycin is an immunosuppressant drug, these animals may be at higher risk for rapamycin toxicity. These toxicities prompted further review, as they have not been observed in our prior studies. As shown in Additional File 7, we noted a lack of weight gain in nude mouse cohorts treated with rapamycin. These toxicities also prompted a comparison of weights before and after treatment in our A/J *Tsc2*^+/- ^experiment; there was no significant difference in weights before and after treatment in the rapamycin treated cohorts and there was no difference in the average weights of the untreated 9 month and 12 month cohorts (see Additional File 3). Although the average weight of one of the rapamycin treated cohorts (Group 2, rapamycin treated daily × 4 weeks) was lower than the untreated group at 12 months (Group 4), the difference was small. We did not observe any increased mortality in the rapamycin treated *Tsc2*^+/- ^cohorts.

## Discussion

The *Tsc2*^+/- ^mouse is an excellent mouse model for the study of TSC related kidney disease. We have previously used *Tsc2*^+/- ^mice in a C57BL/6 mixed strain to show that mTOR inhibitor treatment reduces kidney tumor severity, to investigate the timing of mTOR inhibitor treatment, and to show that addition of prolonged weekly maintenance rapamycin treatment was extremely effective [20,21,61]. However, a major disadvantage of the *Tsc2*^+/- ^mouse model in a predominantly C57BL/6 background is that kidney disease develops gradually so preclinical studies can take 12-18 months to complete. In this study, we sought to improve the *Tsc2*^+/- ^mouse as a preclinical model for TSC tumor studies. Based on observations regarding strain differences reported in Onda et al. 1999 [60], we backcrossed the *Tsc2*^+/- ^genotype onto A/J and C57BL/6 backgrounds, compared kidney disease severity, and found that the A/J strain shows a much higher kidney tumor burden than mice in the C57BL/6 background at 9 and 12 months of age as shown by the average score per kidney and average number of cystadenomas per kidney. Similar to TSC related kidney disease in humans, the tumor burden increases with age in both mouse strains. Interestingly, the A/J *Tsc2*^+/- ^strain shows a significantly higher tumor burden at 5 months of age than the C57BL/6 *Tsc2*^+/- ^strain at 12 months of age. Based on the findings of this study, the A/J strain *Tsc2*^+/- ^mice have a 5-10 fold higher disease burden than C57BL/6 strain *Tsc2*^+/- ^mice and are a superior and higher through-put *Tsc2*^+/- ^mouse model for preclinical studies relevant to TSC kidney disease and tumors. Furthermore, because there is a dramatic difference in the severity of the kidney tumor phenotype in these two mouse strains, they could be used to identify modifier genes that impact the severity of TSC renal manifestations [66].

The potential utility of rapamycin treatment for a prolonged duration was suggested by the results of a previous preclinical study using C57BL/6 *Tsc2*^+/- ^mice in which we noted that a rapamycin dosing schedule that included daily treatment for 2 months and weekly treatment for 6 months, resulted in a dramatic 94.5% reduction in kidney tumor severity [61]. In that study, rapamycin (IP) was given at a dose of 8 mg/kg Monday through Friday from 6 to 7 months of age, followed by a maintenance dose of 16 mg/kg once a week from 7 to 12 months of age, followed by daily rapamycin treatment (8 mg/kg Monday through Friday) from 12 to 13 months of age. We also note that in previous CCI-779 preclinical studies, giving a lower dose over 3 months seemed to be more effective than a higher dose for 2 months (84% reduction with a total dose of 4.32 mg per mouse [21] versus 64% reduction with a total dose of 9.6 mg per mouse [20]. These studies suggest that dosing of mTOR inhibitors at a low dose for a prolonged period of time may be the optimal strategy to maximize benefit and limit drug toxicity. However, a major limitation in understanding the impact of dose intensity, duration of therapy, and weekly mTOR inhibitor dosing based on our prior preclinical studies is that we have previously compared treatment groups from different preclinical studies with important inter-study differences. Because the issue of optimizing rapamycin dosing to maximize efficacy while limiting toxicity has clinical implications, here we further investigated the issue of rapamycin dosing schedule and dose intensity by directly comparing three different rapamycin treatment groups (daily × 4 weeks, daily × 4 weeks then weekly × 8 weeks, and weekly × 12 weeks). We found that optimal treatment correlated with duration of treatment, not total dose given. There was a 66% reduction with a total dose of 4.8 mg per mouse in the group treated daily × 4 weeks, an 82% reduction with a total dose of 6.72 mg per mouse in the group treated daily × 4 weeks plus weekly × 8 weeks, and an 81% reduction with a total dose of 2.88 mg per mouse in the group treated weekly × 12 weeks (see Table 1). These findings demonstrate that low dose rapamycin treatment for a longer duration of time is most effective in the *Tsc2*^+/- ^mouse, and it would be reasonable to evaluate this dosing strategy in future TSC clinical trials.

Our findings also clearly demonstrate that the response of kidney tumors to rapamycin in the *Tsc2*^+/- ^mouse correlates well with observations in early TSC angiomyolipoma clinical trials. In A/J *Tsc2*^+/- ^mice, cystadenoma score per kidney in untreated animals at 9 months of age is 74.4, and cystadenoma score per kidney is 41.13 in the groups treated daily × 4 weeks, but 21.50 in the group treated daily × 4 weeks then weekly × 8 weeks (Table 1). Furthermore, the higher kidney tumor score in the group treated daily × 4 weeks (compared with the group treated daily × 4 weeks then weekly × 8 weeks) is consistent with tumor regrowth during months ~10-12 when no drug treatment was given. This result is analogous to what is observed in patients with kidney angiomyolipomas associated with TSC and/or LAM treated with rapamycin. In a cohort of 20 TSC and/or LAM patients treated with rapamycin for 12 months and then followed off of treatment at 18 months and 24 months, the average kidney angiomyolipoma volume was 71.6 ml at baseline, 36.5 ml at 12 months (~50% size reduction), 64.8 ml at 18 months, and 74.9 ml at 24 months [34]. In both mice and humans, TSC related kidney tumors regress during rapamycin treatment and regrow when rapamycin treatment is stopped. This striking similarity further illustrates the clinical relevance of preclinical studies using the *Tsc2*^+/- ^mouse model. There is also some early evidence that TSC tumor preclinical models are relevant to TSC brain manifestations as several mouse models with TSC related brain abnormalities (seizures or cognitive deficits) also had a reduction of disease severity with rapamycin treatment [67-69].

There is excitement regarding the recent clinical studies showing that rapamycin treatment causes TSC-related tumor regression. However, since regression is incomplete, and tumors regrow with cessation of treatment [32-34] there is significant interest in identifying novel agents for TSC-related tumors to be used either as single agents or in combination with rapamycin. In this study, we evaluated three novel drug classes in our *Tsc2*^-/- ^subcutaneous tumor model: an enzyme that interferes with amino acid metabolism (asparaginase), two VEGF inhibitors (sunitinib and bevacizumab), and a microtubule inhibitor (vincristine). These drugs were tested both as single agents and in combination with rapamycin. We found that asparaginase, sunitinib, and bevacizumab are effective as single agents, but not as effective as rapamycin. Vincristine was not effective as a single agent. None of these drugs combined with rapamycin was more effective than single agent rapamycin treatment. Based on 24 hour rapamycin level measurements, there was no evidence that drug interaction issues influenced the outcome of rapamycin combination treatment with sunitinib or bevacizumab. Rapamycin levels were not tested in the combination groups with asparaginase or vincristine because of the dosing schedule used.

Although asparaginase, sunitinib, and bevacizumab had only a modest improvement (24-27%) in median survival compared to untreated control groups (p values = 0.010-0.019), this difference was statistically significant. In contrast, the improvement in median survival of rapamycin treatment was dramatic (173% compared with untreated, p value = < 0.0001). The positive results with asparaginase treatment are consistent with the known influence of amino acid depletion on the TSC1/TSC2-mTOR signaling pathway [35]. Similarly, the positive results with sunitinib and bevacizumab are consistent with the known relevance of the VEGF signaling pathway in TSC related lesions and in vitro studies of TSC deficient cells [39,40].

There are now several preclinical studies in mouse models of TSC related tumors that have evaluated the efficacy of alternatives to mTOR inhibitors as either single agents or in combination with an mTOR inhibitor. Single agent drugs which are FDA approved for other indications that are effective in mouse TSC tumor models include interferon gamma (IFN-γ), sunitinib, bevacizumab, asparaginase, and tamoxifen. There are also several drugs in development (so are not FDA approved) with single agent activity in TSC tumor models; these include a MEK1/2 inhibitor (CI-1040) [70] and a dual PI3K/mTOR inhibitor (NVP-BEZ-235) [71]. Drugs for which combination with mTOR inhibitor treatment is more effective than single agent mTOR inhibitor include IFN-γ and sorafenib (both are FDA approved for other indications). In order to evaluate optimal strategies for future clinical trials for TSC related tumors, we have reviewed all TSC tumor preclinical studies focusing on results that included positive findings with non-mTOR inhibitors. As many were done using the *Tsc2*^-/- ^subcutaneous tumor model, we have summarized the results from this model in Table 4 from this and previous studies [20,21,31,61]. This summary shows that mTOR inhibitors are clearly most effective with improvements in median survival ranging from 52-173%. The combination of IFN-γ plus CCI-779 improved median survival over untreated by 220% compared with 134% for single agent CCI-779. The combination of sorafenib plus rapamycin improved median survival over untreated by 134% compared with 88% for single agent rapamycin. Single agent drug treatment alternatives to mTOR inhibitors improved median survival from 24-52% (IFN-γ, sunitinib, bevacizumab and asparaginase). Tamoxifen was used to treat *Tsc1*^+/- ^mice (in 129/sv background) and was found to reduce the frequency and severity of liver hemangiomas [72]. It is encouraging to note that there is limited case report evidence that treatment of TSC related tumors with tamoxifen may also correlate with findings in mouse models. There is one report of a massive liver angiomyolipoma in a 26 year old female with *TSC2 *disease that regressed after treatment with tamoxifen [73]. The MEK1/2 inhibitor was used to treat estrogen induced tumors derived from *Tsc2*-null uterine leiomyoma cells. In this model, the mTOR inhibitor RAD001 completely blocked both primary tumor growth and lung metastasis, and a MEK1/2 inhibitor (CI-1040) inhibited lung metastasis. The MEK1/2 inhibitor also partially inhibited primary tumor growth but this was not statistically significant and not as effective as the mTOR inhibitor [70]. The dual PI3K/mTOR inhibitor (NVP-BEZ-235) was used to treat ENU-accelerated kidney tumors in the *Tsc2*^+/- ^mouse. Although NVP-BEZ-235 reduced the severity of kidney disease to a similar degree as RAD001, the combination of RAD001 plus NVP-BEZ-235 was similar to single agents [71]. There are also several drugs that were not effective in preclinical models including vincristine, doxycycline, and atorvastatin [61,74].

**Table 4:** Summary of Survival Data for Effective Agents in the *Tsc2-/- *Subcutaneous Tumor Model

Reference	Start Criteria	Treatment Cohort	Dosing	Median Survival (days)	Percent Difference From Untreated	Drug class
Current Study	Tumor Volume of 100 mm3	Untreated	-	31	-	
		Rapamycin	8 mg/kg 3 days/wk	84.5	173%	*
		Asparaginase	2IU/g twice/wk × 4 wks	39.5	27%	#
		Asparaginase + Rapamycin	2IU/g twice/wk × 4 wks + 8 mg/kg 3 days/wk	71	129%	
		Sunitinib	80 mg/kg 5 days/wk	39	26%	#
		Sunitinib + Rapamycin	80 mg/kg 5 days/wk + 8 mg/kg 3 days/wk	80	158%	
		Bevacizumab	5 mg/kg once/2 wks	38.5	24%	#
		Bevacizumab + Rapamycin	5 mg/kg once/2 wks + 8 mg/kg 3 days/wk	60	94%	

Lee et al, 2009	Tumor Volume of 150 mm3	Untreated	-	24.5	-	
		Rapamycin	8 mg/kg 5 days/wk	46	88%	*
		Sorafenib	60 mg/kg 5 days/wk	19.5	-20%	
		Sorafenib + Rapamycin	60 mg/kg 5 days/wk + 8 mg/kg 5 days/wk	53	116%	**

Messina et al, 2007	Tumor Volume of 50 mm3 for early treatments, 250 mm3 for late treatments	Untreated	-	31	-	
		Early CCI-779	8 mg/kg 5 days/wk	47	52%	*
		Early Rapamycin	8 mg/kg 5 days/wk	62	100%	*
		Late Rapamycin	8 mg/kg 5 days/wk	59	90%	*

Lee et al, 2006	Tumor Volume of 300 mm3	Untreated	-	17.5	-	
		CCI-779	8 mg/kg 5 days/wk	41	134%	*
		IFN-γ	20,000 units 3 days/wk	22	26%	#
		IFN-γ + CCI-779	20,000 units 3 days/wk + 8 mg/kg 5 days/wk	56	220%	**

Lee et al, 2005	18 Days after injection with *Tsc2-/- *cells	Untreated	-	33	-	
		CCI-779	4 mg/kg 3 days/wk	69	109%	*
		IFN-γ	20,000 units 3 days/wk	50	52%	#

* Single agent mTOR inhibitor (all agents and doses were more effective than no treatment)

** Combination containing mTOR inhibitor that was more effective than single agent mTOR inhibitor

# Single agent other than mTOR inhibitor that was more effective than no treatment

## Conclusions

The preclinical studies reported here show that the A/J *Tsc2*^+/- ^mouse model has younger onset TSC related kidney disease and as a result, is an improved mouse model for use in future preclinical studies. Our rapamycin dosing comparison results in A/J *Tsc2*^+/- ^mice indicate that a longer duration of rapamycin treatment is more important than dose intensity, therefore low doses for a prolonged duration seems to be the best strategy. Since the response to mTOR inhibitors in *Tsc2*^+/- ^mice correlates well with observations in rapamycin kidney angiomyolipoma trials, it would be reasonable to test this dosing strategy in future TSC clinical trials. We also present data showing evidence for tumor response to some new single agents including sunitinib, bevacizumab, and asparaginase. We have previously shown that single agent IFN-γ, combination IFN-γ plus mTOR inhibitor, and combination sorafenib plus mTOR inhibitor are effective in the *Tsc2*^-/- ^subcutaneous tumor model. Since tumor responses to mTOR inhibitor treatment are much more dramatic than responses to other agents (see Table 4) and combination treatments are only a slight improvement over single agent mTOR inhibitor treatment, single agent mTOR inhibitor treatment seems to be the best initial strategy for medical treatment of problematic TSC related tumors. We conclude that clinical investigation of non-mTOR inhibitors as single agents or in combination with an mTOR inhibitor should be investigated as second line therapy for problematic TSC related tumors that are not responding to mTOR inhibitors. This work illustrates the clinical relevance of preclinical studies in mouse models of *TSC2 *related tumors. Future preclinical studies using these and related mouse models are likely to guide a rational approach to improving medical therapy for TSC related tumors and other manifestations of TSC.

## Competing interests

The authors declare that they have no competing financial interests. SD is the overall Principal Investigator on a multi-center trial evaluating the efficacy and safety of rapamycin for the treatment of kidney angiomyolipomas http://www.clinicaltrials.gov/ct2/show/NCT00126672. This is an investigator initiated trial funded by the National Institutes of Health (National Cancer Institute) and the Tuberous Sclerosis Alliance. Wyeth is providing free study drug but no funding. SD also holds a patent (not licensed) on the use of IFN-γ (Interferon Gamma in the Detection and Treatment of Angiomyolipomas, US patent 7,229,614).

## Authors' contributions

CW assisted with experimental design, performed data collection and statistical analyses, and wrote and helped edit the manuscript.

AN assisted with experimental design, performed data collection and statistical analyses, and wrote and helped edit the manuscript.

SD provided funding, critical guidance for the experiments, and was responsible for supervising the writing and editing of the manuscript.

All authors have read and approved this manuscript.

## Supplementary Material

Additional file 1 **Tumor Scoring Scale**. Table showing tumor scoring scale. Click here for file

Additional file 2 **Kidney Lesion Type Scale**. Table with definition of kidney cystadenoma subtypes. Click here for file

Additional file 3 **No Difference in Weight at the Beginning and End of Treatment in A/J *Tsc2*^+/- ^Mice**. Table with average weight data for cohorts of A/J *Tsc2*^+/- ^mice. Click here for file

Additional file 4 **Summary of Toxicities in Mice with *Tsc2*^-/- ^Subcutaneous Tumors**. Table summarizing mice with *Tsc2*^-/- ^subcutaneous tumors mice that required euthanasia due to toxicity. Click here for file

Additional file 5 **There is no difference in severity of kidney disease between untreated males and females in both the A/J *Tsc2*^+/- ^and the C57BL/6 *Tsc2*^+/- ^strains**. Figure showing the average score per kidney for each cohort. The p-values compare males and females within the same strain at a specific time point (either nine or twelve months of age). None of the p-values indicate a statistical difference (p < 0.05). Click here for file

Additional file 6 **Bevacizumab and sunitinib do not significantly affect whole blood rapamycin levels in nude mice bearing *Tsc2*^-/- ^tumors**. Figure showing whole blood rapamycin levels from indicated treatment groups. Rapamycin levels were measured 24 hours after the last dose of rapamycin for all groups. Click here for file

Additional file 7 **Failure to Gain Weight in Mice with *Tsc2*^-/- ^Subcutaneous Tumors Treated with Rapamycin**. Table showing lack of weight gain in mice with *Tsc2*^-/- ^subcutaneous tumors treated with rapamycin. Click here for file
