# Supplementary material for: Comparison of three rapamycin dosing schedules in A/J Tsc2+/- mice and improved survival with angiogenesis inhibitor or asparaginase treatment in mice with subcutaneous tuberous sclerosis related tumors
Source: J Transl Med. 2010 Feb 10;8:14. doi: 10.1186/1479-5876-8-14 (PMC2834646; doi:10.1186/1479-5876-8-14)
Supplement: Additional file 1 — Tumor Scoring Scale. Table showing tumor scoring scale. [file 1479-5876-8-14-S1.PDF]

Additional File 1
Title: Tumor Scoring Scale

Score	Area Range (mm^2)
0	0
1	$0 < x \le 0.09$
2	$0.09 < x \le 0.2$
3	$0.2 < x \le 0.35$
4	$0.35 < x \le 0.5$
5	$x > 0.5$
