# Supplementary material for: Comparison of three rapamycin dosing schedules in A/J Tsc2+/- mice and improved survival with angiogenesis inhibitor or asparaginase treatment in mice with subcutaneous tuberous sclerosis related tumors
Source: J Transl Med. 2010 Feb 10;8:14. doi: 10.1186/1479-5876-8-14 (PMC2834646; doi:10.1186/1479-5876-8-14)
Supplement: Additional file 2 — Kidney Lesion Type Scale. Table with definition of kidney cystadenoma subtypes. [file 1479-5876-8-14-S2.PDF]

Additional File 2
Title: Kidney Lesion Type Definition

Category	Characteristics
Cyst	0% filled
Pre-Papillary	>0% and ≤25% filled
Papillary	>25% and <100% filled
Solid	100% filled
