# Supplementary material for: Comparison of three rapamycin dosing schedules in A/J Tsc2+/- mice and improved survival with angiogenesis inhibitor or asparaginase treatment in mice with subcutaneous tuberous sclerosis related tumors
Source: J Transl Med. 2010 Feb 10;8:14. doi: 10.1186/1479-5876-8-14 (PMC2834646; doi:10.1186/1479-5876-8-14)
Supplement: Additional file 3 — No Difference in Weight at the Beginning and End of Treatment in A/J Tsc2+/- Mice. Table with average weight data for cohorts of A/J Tsc2+/- mice. [file 1479-5876-8-14-S3.PDF]

Additional file 3

Title: No Difference in Weight at the Beginning and End of Treatment in A/J *Tsc2* $^{+/-}$ Mice

	Weight at Start of Treatment (g) (9 months of age) (ave ± std error)	p value vs. untreated (9 months of age)	Weight at End of Treatment (g) (12 months of age) (ave ± std error)	p value vs. untreated (12 months of age)	Number of Mice
Group 1<br>A/J rapa daily x 4 weeks then weekly x 8 weeks	30.83 ± 2.90	0.5041	27.88 ±1.36	0.3422	8
Group 2<br>A/J rapa daily x 4 weeks	27.91 ± 1.85	0.353	26.69 ± 0.94	0.0526	8
Group 3<br>A/J rapa weekly x 12 weeks	29.50 ± 1.24	0.9587	28.37 ± 0.88	0.4153	9
Group 4<br>A/J, untreated, 12 months	not available	0.9874	29.41 ± 0.88	n/a	8
A/J, untreated, 9 months	29.43 ± 0.70	n/a	not available	0.9874	16
