# Supplementary material for: Comparison of three rapamycin dosing schedules in A/J Tsc2+/- mice and improved survival with angiogenesis inhibitor or asparaginase treatment in mice with subcutaneous tuberous sclerosis related tumors
Source: J Transl Med. 2010 Feb 10;8:14. doi: 10.1186/1479-5876-8-14 (PMC2834646; doi:10.1186/1479-5876-8-14)
Supplement: Additional file 4 — Summary of Toxicities in Mice with Tsc2-/- Subcutaneous Tumors. Table summarizing mice with Tsc2-/- subcutaneous tumors mice that required euthanasia due to toxicity. [file 1479-5876-8-14-S4.PDF]

Additional File 4
Title: Summary of Toxicities in Mice with *Tsc2*$^{-/-}$ Subcutaneous Tumors

Mice Euthanized Early due to Toxicity		
**Treatment Group**	**Day**	**Tumor Volume (mm^3)**
Rapamycin	85	1715
Vincristine+Rapamycin	98	2176
Asparaginase+Rapamycin	70	1366
Asparaginase+Rapamycin	84	1563
Sunitinib+Rapamycin	39	2250
Bevacizumab+Rapamycin	66	1856
