# Supplementary figures and images for: Comparison of three rapamycin dosing schedules in A/J Tsc2+/- mice and improved survival with angiogenesis inhibitor or asparaginase treatment in mice with subcutaneous tuberous sclerosis related tumors
Source: J Transl Med. 2010 Feb 10;8:14. doi: 10.1186/1479-5876-8-14 (PMC2834646; doi:10.1186/1479-5876-8-14)

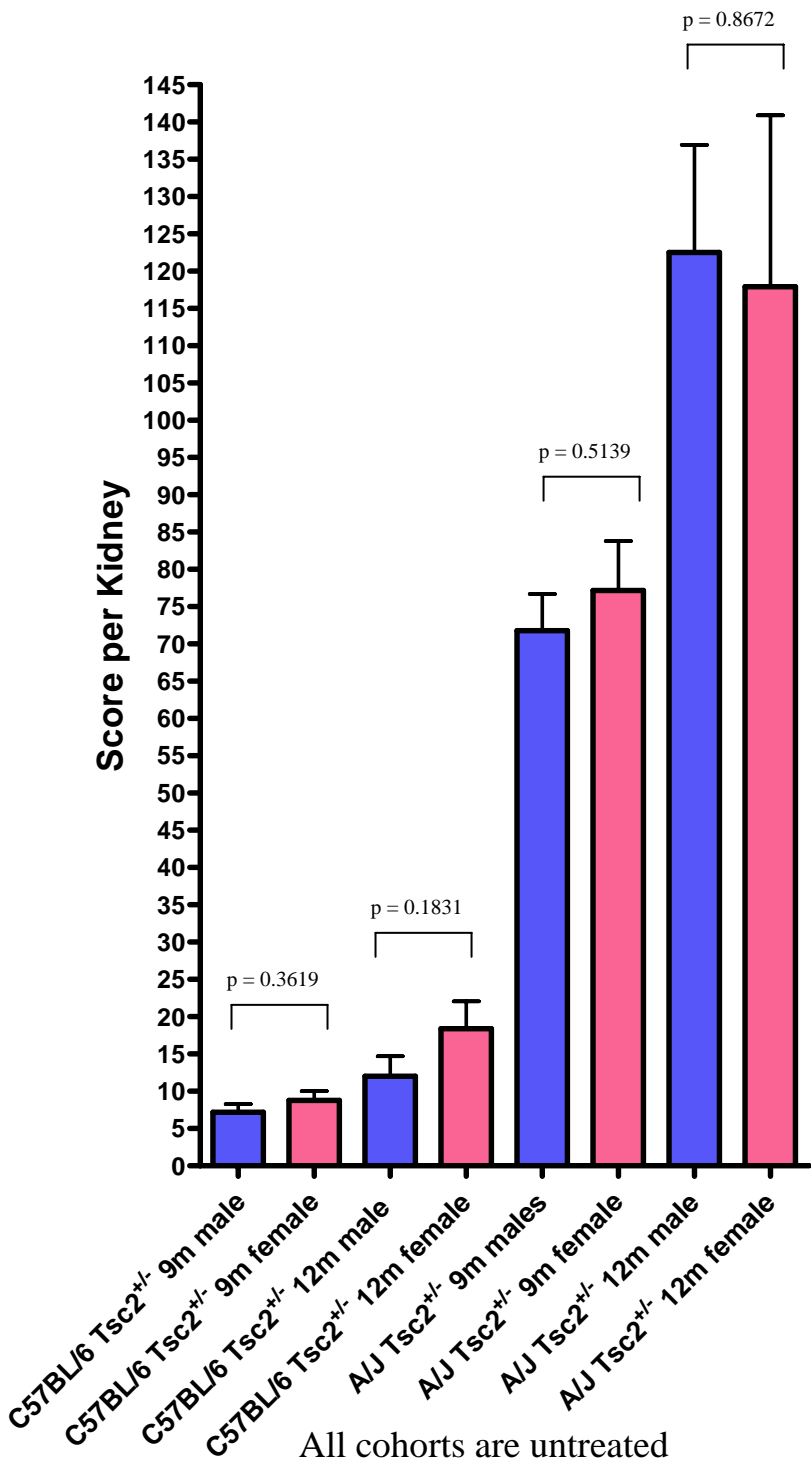

Supplement: Additional file 5 — There is no difference in severity of kidney disease between untreated males and females in both the A/J Tsc2+/- and the C57BL/6 Tsc2+/- strains. Figure showing the average score per kidney for each cohort. The p-values compare males and females within the same strain at a specific time point (either nine or twelve months of age). None of the p-values indicate a statistical difference (p < 0.05). [file 1479-5876-8-14-S5.PDF]

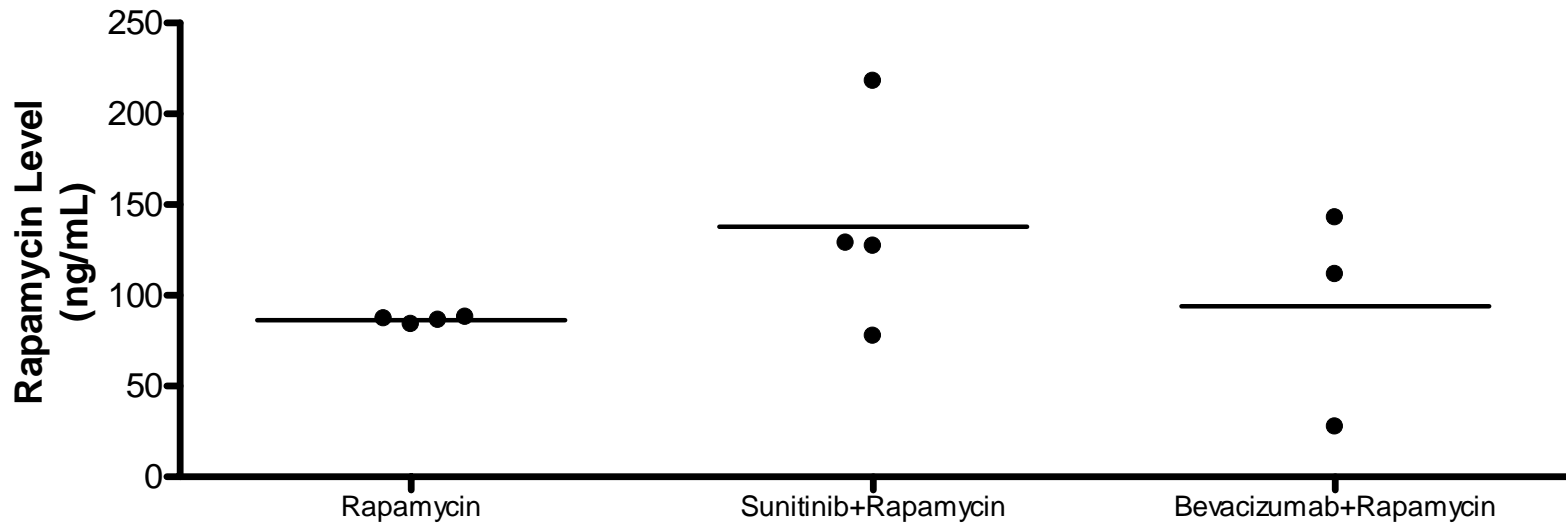

Supplement: Additional file 6 — Bevacizumab and sunitinib do not significantly affect whole blood rapamycin levels in nude mice bearing Tsc2-/- tumors. Figure showing whole blood rapamycin levels from indicated treatment groups. Rapamycin levels were measured 24 hours after the last dose of rapamycin for all groups. [file 1479-5876-8-14-S6.PDF]
