# Supplementary material for: Comparison of three rapamycin dosing schedules in A/J Tsc2+/- mice and improved survival with angiogenesis inhibitor or asparaginase treatment in mice with subcutaneous tuberous sclerosis related tumors
Source: J Transl Med. 2010 Feb 10;8:14. doi: 10.1186/1479-5876-8-14 (PMC2834646; doi:10.1186/1479-5876-8-14)
Supplement: Additional file 7 — Failure to Gain Weight in Mice with Tsc2-/- Subcutaneous Tumors Treated with Rapamycin. Table showing lack of weight gain in mice with Tsc2-/- subcutaneous tumors treated with rapamycin. [file 1479-5876-8-14-S7.PDF]

Additional File 7
Title: Failure to Gain Weight in Mice with Tsc2-/- Subcutaneous Tumors Treated with Rapamycin

Group	Weight at Start (g) (ave ± std error)	p value vs. untreated at start	Weight at Sac (g) (ave ± std error)	p value vs. untreated at sac	Change in Weight (g) (ave ± std error)	Number of Mice
Untreated	33.65 ± 0.79	-	37.78 ± 0.70	-	4.125 ± 0.69	8
Rapamycin Treated	33.74 ± 0.39	NS	32.98 ± 0.56	0.0005	-0.8132 ± 0.58	34
Other Treatment	34.61 ± 0.55	NS	35.53 ± 0.73	NS	0.9188 ± 0.89	32
